# Metabolites mediate the effects of healthy lifestyles on the risks of common age-related diseases

**DOI:** 10.3389/fendo.2025.1654172

**Published:** 2025-10-17

**Authors:** Yan Li, Huijuan Li, Xiaoyu Chen, Xueyan Liang

**Affiliations:** ^1^ Department of Clinical Pharmacy, Guangxi Academy of Medical Sciences and the People’s Hospital of Guangxi Zhuang Autonomous Region, Nanning, China; ^2^ Phase I Clinical Trial Laboratory, Guangxi Academy of Medical Sciences and the People’s Hospital of Guangxi Zhuang Autonomous Region, Nanning, China

**Keywords:** healthy lifestyle, metabolites, age-related disease, diabetes, chronic diseases

## Abstract

**Background:**

Limited research is available on the associations between healthy lifestyles and age-related diseases, particularly those involving multiple diseases and their underlying mechanisms. We aimed to determine whether healthy lifestyles are associated with a lower likelihood of age-related diseases, and whether metabolites mediate these associations.

**Methods:**

The UK Biobank data cohort was used in this study. Five lifestyle factors (diet, physical activity, sedentary behavior, sleep duration, and alcohol consumption) were combined to determine that composite lifestyle scores. Lifestyle-related metabolic signatures were analyzed using Cox proportional hazards models. We then conducted sequential analyses combining Cox regression, linear regression, extreme gradient boosting (XGBoost), and Shapley additive explanation (SHAP) values to identify metabolites associated with age-related diseases and healthy lifestyle scores. Mediation analysis was performed to investigate the potential mediating effects of the identified metabolites on age-related diseases.

**Results:**

Healthy lifestyle scores contributed the most to prevention of chronic obstructive pulmonary disease (COPD) (hazard ratio [HR] [95% confidence interval (CI): 0.72 (0.71, 0.74)], followed by emphysema [HR (95% CI): 0.75 (0.71, 0.78)]. Furthermore, intermediate or healthy lifestyles significantly decreased the age-related risk of stroke, chronic liver disease, chronic kidney disease (CKD), osteoporosis, osteoarthritis, and hypertension. Age-related diseases were associated with the top 10 metabolites, and these associations were individually or jointly mediated. For example, glycoprotein acetylation contributed 14.43% to the overall association between healthy lifestyle scores and inflammatory bowel disease (IBD), whereas low-density lipoprotein (LDL) cholesterol level attenuated this association by 2.92%, the fatty acid content based on the degree of unsaturation showed a 21.64% contribution to the association between the healthy lifestyle score and type 2 diabetes, whereas cholesterol esters in large high-density lipoproteins (HDLs) accounted for 4.57%. Sensitivity analyses verified the robustness and validity of these findings.

**Conclusions:**

These findings provide a deeper understanding of the intricate relationships among lifestyle and metabolites and the development of age-related diseases.

## 1 Introduction

Most chronic diseases and deaths are associated with age (1, 2). In old age, the body loses its functionality and integrity, which leads to the development of major diseases, which often co-occur and lead to death. The incidence of major chronic diseases, including stroke, diabetes, ischemic heart disease, kidney and liver diseases, and a variety of other conditions are increasing (2, 3). From 1990 to 2017, the percentage of people aged 65 and older increased from 6.1% to 8.8%. This demographic change has contributed to an additional 12 million deaths worldwide (4). The aging global population is burdened by the increasing prevalence of age-related diseases, which pose both economic and health-related burdens. Identifying interventions that promote healthy aging is crucial for preventing chronic conditions. Although digestive conditions such as inflammatory bowel disease (IBD) do not directly contribute to mortality risk, they are common conditions and have a major influence on healthcare and economics (5). Recent evidence from large-scale proteomics studies has demonstrated that biological aging, as measured using circulating protein signatures, is strongly associated with the incidence of multiple chronic diseases and multimorbidities (6). In particular, a proteomic aging clock developed using the UK Biobank cohort was shown to accurately predict the onset of major age-related diseases, reinforcing the idea that these conditions share common age-related biological pathways (6). Therefore, evaluation of the major modifiable factors associated with these chronic age-related conditions is of great importance.

Healthy lifestyles appear to reduce chronic disease risks as well as mortality risks and promote healthy aging, according to previous studies (7–10). A healthy lifestyle is important for chronic conditions, highlighting the importance of consuming alcohol within permitted limits, following a healthy diet, and exercising regularly. These lifestyle factors are vital for preventing and reversing chronic conditions and can be effectively targeted (11–15). Large-scale cohort studies have supported these findings. For example, a previously published study demonstrated that adherence to multiple healthy lifestyle factors, including nonsmoking, moderate alcohol intake, regular physical activity, and a balanced diet, was associated with a substantially lower risk of type 2 diabetes among Chinese adults (16). Similarly, another study found that a composite healthy lifestyle score integrating diet, physical activity, smoking status, and body weight was inversely associated with the incidence of cardiovascular disease in a Spanish population (17, 18). The combined effects of some new lifestyle factors, including adequate sleep duration and non-sedentary behavior, on chronic disease risk have also been identified (19–22). Comparisons of individual lifestyle factors with combinations of these behaviors can reveal how they perform on a daily basis and interact synergistically (23). Although lifestyle behavioral effects can be accurately evaluated, several studies were limited by the use of self-reported data, which are prone to measurement and recall biases. Due to biological heterogeneity, internal metabolic responses to all healthy behaviors, including dietary choices, are likely to vary significantly (24).

The use of Nuclear Magnetic Resonance (NMR) spectroscopy in metabolomics has become increasingly popular owing to its remarkable features, such as high reproducibility, quantitative capabilities, non-selective, and non-invasive nature, as well as the ability to identify unknown metabolites in complex mixtures (25). Metabolic profiling using high-throughput technology has shown substantial promise for assessing the metabolic responses of individuals to genetic and lifestyle variables (26). In addition to measuring a multitude of essential biomarkers, metabolic profiles can detect changes within relevant biological pathways, enabling objective assessment of complex lifestyle patterns (27, 28). Various metabolic signatures have been identified for lifestyle behaviors and their associations with various conditions, including myocardial infarction, stroke, coronary artery disease, and cancers, have been extensively explored (29–31). However, only a few studies have examined the relationship between metabolic signatures and incident age-related diseases.

Thus, we hypothesized that a healthy lifestyle may contribute to a decline in age-related diseases by influencing the levels of certain metabolites. Using metabolomics, small molecules (metabolites) in biological samples can be comprehensively analyzed, providing valuable insights into the dynamic metabolic processes influenced by healthy lifestyle choices. In the present study, by analyzing data from the UK Biobank, we examined the association between adherence to a healthy lifestyle and 12 age-related diseases. Another objective of this study was to evaluate the associations of 168 metabolites identified using NMR with the risk of each age-related disease and to examine whether these identified metabolites play a mediating role in the associations between healthy lifestyle scores and age-related disease risk.

## 2 Methods

### 2.1 Ethics statement

In accordance with the Declaration of Helsinki, all participants provided written informed consent prior to enrollment. The study protocol was reviewed and approved by the NHS National Research Ethics Service (Ref: 11/NW/0382).

### 2.2 Participant information

The study population was identified from the UK Biobank, which collects and stores biological samples as well as physiological, pathological, socioeconomic, and other information about participants. More than 500,000 participants from 22 assessment centers in England, Scotland, and Wales participated in the UK Biobank study between 2006 and 2010. A follow-up study was conducted in England until October 31, 2022, in Scotland until August 31, 2022, and in Wales until May 31, 2022.

### 2.3 Disease detection

Twelve age-related diseases were analyzed, IBD, type 2 diabetes, stroke, ischemic heart disease, chronic obstructive pulmonary disease (COPD), emphysema, chronic liver disease, chronic kidney disease (CKD), osteoporosis, osteoarthritis, hypertension, and obesity. All age-related diseases were categorized according to International Classification of Diseases (ICD) diagnosis codes, with the corresponding dates of diagnosis obtained from the linked hospital inpatient, primary care, and death registers. Using the UK Biobank lookup table, primary care read codes were converted into ICD codes. The UK Biobank data portal provides access to inpatient hospital records, primary care data, and cancer register data as of December 14, 2024. **Supplementary Table 1** contains the ICD-10 and ICD-9 codes related to the age-related diseases evaluated in this study. Participants were considered to have a disease at baseline if they reported that they had ever been diagnosed with it, received medication, or had a pre-existing ICD diagnosis. To define the date of disease onset, we used the earliest available date recorded in the medical records. To enhance the statistical power, participants were excluded from the analysis of from each disease group if they showed the corresponding disease at baseline. The follow-up period for each participant was calculated from the date of the first assessment until the date of the first diagnosis of the study outcome, death, loss to follow-up, or end of follow-up, whichever came first.

### 2.4 Scores for healthy lifestyles

On the basis of previous research, we selected five modifiable factors that contribute to a healthy lifestyle score (32). These included three conventional factors, namely, alcohol consumption, physical activity, and diet quality, as well as two emerging factors, namely, sleep duration and sedentary behavior. Each healthy behavior received a point, and an overall lifestyle that ranging from 0 to 5 was assessed on the basis of the cumulative sum of the scores for these factors. Higher lifestyle scores indicated healthier lifestyles. On the basis of the overall score, lifestyle was categorized as healthy (score, 4-5), intermediate (score, 2-3), or poor (score, 0-1) (33–35). **Supplementary Table 2** provides a detailed description of the methods and definitions used to collect data from the baseline assessment, including the touchscreen questionnaire.

Health promoting behavior was defined as consumption of no more than 14 and 28g of alcohol per day by women and men, respectively, based on the maximum limits set by the US dietary guidelines (36). Fitness for well-being was defined as 150 min of moderate or 75 mins of vigorous or equivalent physical activity per week (37). Diets that included at least half of the following eight criteria were considered healthy: eating more fruits, more vegetables, more whole grains, and more fish, less refined grains, less processed meats, less unprocessed red meat, and fewer sugary foods or beverages (38, 39). Individuals who spend less than 2 h a day watching television or using a computer (excluding work) were considered to be less likely to engage in sedentary behaviors (39). Six to eight hours of sleep per day were considered adequate (39).

### 2.5 Measurement of metabolites

Ethylenediaminetetraacetic acid (EDTA) plasma samples from a random subset of approximately 280,000 UK Biobank participants were analyzed using a high-throughput NMR-metabolomics platform obtained from Nightingale Health. Currently, data are available for phases 1 and 2 of this study. The original 168 metabolites present in the samples, including fatty acids, glycolytic metabolites, ketones, amino acids, lipids, and lipoproteins, were expressed as absolute concentrations (mmol/L). As shown in **Supplementary Table 3**, detailed coding was performed for 168 NMR metabolites from the UK Biobank. The methods used to collect samples and quantify metabolomics in previous studies have been previously described in detail (40).

### 2.6 Covariates

Through questionnaires, sociodemographic factors associated with age-related disease risk were assessed as potential confounders (**Table 1**) (31, 37). By examining factors such as income, employment, and access to services, the Townsend Deprivation Index measures material well-being, with a higher score indicating greater deprivation.

**Table 1:** Baseline characteristics of participants in this study investigating the association of lifestyle metabolites and age-related diseases across lifestyle category.

Characteristic	Participants with complete lifestyle data				Participants with complete lifestyle and metabolites data			
	Overall	Poor	Intermediate	Healthy	Overall	Poor	Intermediate	Healthy
	(n = 293,111)	(n = 19,443)	(n = 180,178)	(n = 93,490)	(n = 164,388)	(n = 11,058)	(n = 101,275)	(n = 52,055)
Age at recruitment, median (IQR)	56 (48, 62)	56 (49, 62)	56 (48, 62)	56 (48, 62)	56 (48, 62)	56 (49, 62)	56 (48, 62)	56 (48, 62)
Follow-up, years, median (IQR)	13.72 (13.03, 14.37)	13.62 (12.85, 14.33)	13.70 (13.01, 14.37)	13.75 (13.09, 14.39)	13.76 (13.05, 14.45)	13.70 (12.87, 14.42)	13.75 (13.04, 14.44)	13.79 (13.11, 14.46)
Sex, n (%)								
Female	161,287 (55%)	9,828 (51%)	95,469 (53%)	55,990 (60%)	90,074 (55%)	5,593 (51%)	53,413 (53%)	31,068 (60%)
Male	131,824 (45%)	9,615 (49%)	84,709 (47%)	37,500 (40%)	74,314 (45%)	5,465 (49%)	47,862 (47%)	20,987 (40%)
Ethnicity, n (%)								
Non-white	12,660 (4.3%)	590 (3.0%)	7,186 (4.0%)	4,884 (5.2%)	6,604 (4.0%)	310 (2.8%)	3,798 (3.8%)	2,496 (4.8%)
White	280,451 (96%)	18,853 (97%)	172,992 (96%)	88,606 (95%)	157,784 (96%)	10,748 (97%)	97,477 (96%)	49,559 (95%)
Education, n (%)								
College or above	140,905 (48%)	7,565 (39%)	82,441 (46%)	50,899 (54%)	78,151 (48%)	4,218 (38%)	45,868 (45%)	28,065 (54%)
High school or equivalent	116,745 (40%)	8,607 (44%)	74,294 (41%)	33,844 (36%)	65,785 (40%)	4,945 (45%)	41,854 (41%)	18,986 (36%)
Less than high school	35,461 (12%)	3,271 (17%)	23,443 (13%)	8,747 (9.4%)	20,452 (12%)	1,895 (17%)	13,553 (13%)	5,004 (9.6%)
Townsend deprivation index, mean (SD)	-1.48 (2.96)	-1.13 (3.13)	-1.47 (2.96)	-1.57 (2.91)	-1.52 (2.95)	-1.15 (3.13)	-1.51 (2.95)	-1.62 (2.89)
Household income								
Equal or high 30,999£	163,900 (56%)	9,895 (51%)	99,890 (55%)	54,115 (58%)	90,426 (55%)	5,535 (50%)	55,120 (54%)	29,771 (57%)
Low 30,999£	129,211 (44%)	9,548 (49%)	80,288 (45%)	39,375 (42%)	73,962 (45%)	5,523 (50%)	46,155 (46%)	22,284 (43%)
Smoking status, n (%)								
Never	205,404 (70%)	10,685 (55%)	122,355 (68%)	72,364 (78%)	115,126 (70%)	6,055 (55%)	68,797 (68%)	40,274 (78%)
Previous	65,477 (22%)	5,711 (29%)	42,558 (24%)	17,208 (18%)	36,649 (22%)	3,258 (30%)	23,767 (24%)	9,624 (19%)
Current	21,649 (7.4%)	3,006 (15%)	14,908 (8.3%)	3,735 (4.0%)	12,284 (7.5%)	1,720 (16%)	8,505 (8.4%)	2,059 (4.0%)
HbA1c, mean (SD), %	35.01 (5.09)	35.36 (5.80)	35.05 (5.17)	34.87 (4.76)	35.02 (5.13)	35.36 (5.79)	35.06 (5.22)	34.86 (4.80)
SBP, mean (SD), mmHg	136.64 (18.51)	139.24 (18.67)	137.28 (18.48)	134.87 (18.37)	136.71 (18.48)	139.41 (18.65)	137.36 (18.45)	134.87 (18.35)
WHR, mean (SD), %	0.86 (0.09)	0.89 (0.09)	0.87 (0.09)	0.85 (0.08)	0.86 (0.09)	0.89 (0.09)	0.87 (0.09)	0.85 (0.08)
BMI category (kg/m^2^), n (%)								
Underweight (<18.5)	1,653 (0.6%)	110 (0.6%)	873 (0.5%)	670 (0.7%)	893 (0.5%)	61 (0.6%)	478 (0.5%)	354 (0.7%)
Normal weight (18.5 to <25)	106,030 (36%)	5,079 (26%)	59,603 (33%)	41,348 (44%)	59,101 (36%)	2,866 (26%)	33,351 (33%)	22,884 (44%)
Overweight (25 to <30)	124,383 (42%)	8,237 (42%)	78,921 (44%)	37,225 (40%)	70,032 (43%)	4,665 (42%)	44,516 (44%)	20,851 (40%)
Obese ≥30	61,045 (21%)	6,017 (31%)	40,781 (23%)	14,247 (15%)	34,362 (21%)	3,466 (31%)	22,930 (23%)	7,966 (15%)
Central obesity, n (%)	86,317 (29%)	8,320 (43%)	57,027 (32%)	20,970 (22%)	48,275 (29%)	4,709 (43%)	31,938 (32%)	11,628 (22%)
Lifestyle factors, n (%)								
Alcohol consumption								
Favorable	201,617 (69%)	3,390 (17%)	109,783 (61%)	88,444 (95%)	113,106 (69%)	1,918 (17%)	61,843 (61%)	49,345 (95%)
Unfavorable	91,494 (31%)	16,053 (83%)	70,395 (39%)	5,046 (5.4%)	51,282 (31%)	9,140 (83%)	39,432 (39%)	2,710 (5.2%)
Physical activity								
Favorable	213,011 (73%)	3,119 (16%)	119,606 (66%)	90,286 (97%)	119,244 (73%)	1,797 (16%)	67,185 (66%)	50,262 (97%)
Unfavorable	80,100 (27%)	16,324 (84%)	60,572 (34%)	3,204 (3.4%)	45,144 (27%)	9,261 (84%)	34,090 (34%)	1,793 (3.4%)
Diet								
Favorable	164,839 (56%)	1,267 (6.5%)	75,933 (42%)	87,639 (94%)	92,050 (56%)	716 (6.5%)	42,573 (42%)	48,761 (94%)
Unfavorable	128,272 (44%)	18,176 (93%)	104,245 (58%)	5,851 (6.3%)	72,338 (44%)	10,342 (94%)	58,702 (58%)	3,294 (6.3%)
Sedentary behavior								
Favorable	37,103 (13%)	117 (0.6%)	9,110 (5.1%)	27,876 (30%)	20,551 (13%)	73 (0.7%)	5,088 (5.0%)	15,390 (30%)
Unfavorable	256,008 (87%)	19,326 (99%)	171,068 (95%)	65,614 (70%)	143,837 (87%)	10,985 (99%)	96,187 (95%)	36,665 (70%)
Sleep duration								
Favorable	260,075 (89%)	9,929 (51%)	157,789 (88%)	92,357 (99%)	145,748 (89%)	5,620 (51%)	88,699 (88%)	51,429 (99%)
Unfavorable	33,036 (11%)	9,514 (49%)	22,389 (12%)	1,133 (1.2%)	18,640 (11%)	5,438 (49%)	12,576 (12%)	626 (1.2%)
No. of healthy lifestyle factors, no. (%)								
0	1,621 (0.6%)	1,621 (8.3%)	0 (0%)	0 (0%)	934 (0.6%)	934 (8.4%)	0 (0%)	0 (0%)
1	17,822 (6.1%)	17,822 (92%)	0 (0%)	0 (0%)	10,124 (6.2%)	10,124 (92%)	0 (0%)	0 (0%)
2	68,313 (23%)	0 (0%)	68,313 (38%)	0 (0%)	38,437 (23%)	0 (0%)	38,437 (38%)	0 (0%)
3	111,865 (38%)	0 (0%)	111,865 (62%)	0 (0%)	62,838 (38%)	0 (0%)	62,838 (62%)	0 (0%)
4	80,848 (28%)	0 (0%)	0 (0%)	80,848 (86%)	45,088 (27%)	0 (0%)	0 (0%)	45,088 (87%)
5	12,642 (4.3%)	0 (0%)	0 (0%)	12,642 (14%)	6,967 (4.2%)	0 (0%)	0 (0%)	6,967 (13%)
Family history, n (%)								
Heart disease	117,885 (45%)	7,789 (45%)	72,215 (45%)	37,881 (45%)	66,582 (45%)	4,467 (45%)	40,796 (45%)	21,319 (46%)
Stroke	73,225 (28%)	4,857 (28%)	44,988 (28%)	23,380 (28%)	41,047 (28%)	2,742 (28%)	25,326 (28%)	12,979 (28%)
Hypertension	139,906 (53%)	8,883 (51%)	85,669 (53%)	45,354 (54%)	78,108 (53%)	5,031 (51%)	47,865 (53%)	25,212 (54%)
Diabetes	60,078 (23%)	4,004 (23%)	37,245 (23%)	18,829 (22%)	33,804 (23%)	2,287 (23%)	20,957 (23%)	10,560 (23%)
Alzheimers	33,822 (13%)	2,322 (13%)	20,629 (13%)	10,871 (13%)	18,739 (13%)	1,303 (13%)	11,384 (13%)	6,052 (13%)
Parkinsons	11,639 (4.4%)	755 (4.3%)	6,952 (4.3%)	3,932 (4.7%)	6,425 (4.4%)	438 (4.4%)	3,855 (4.2%)	2,132 (4.6%)

BMI, body mass index; HbA1c, glycated haemoglobin; SBP, systolic blood pressure; WHR, waist-hip ratio. Values for categorical variables are presented as number (percentage). Age at recruitment and follow-up time are presented as median (interquartile range [IQR]). All other continuous variables are presented as mean (standard deviation [SD]).

### 2.7 Statistical analyses

#### 2.7.1 Diseases associated with aging and healthy lifestyles

Using Cox proportional hazard regression models to adjust for potential confounding variables, healthy lifestyle scores and categories were analyzed to evaluate their associations with the incidence of each of the 12 age-related diseases. Three models were tested in the study. Model 1 included no adjustments. Model 2 included adjustments for age, sex, racial or ethnic background, education, household income, and Townsend Deprivation Index. Model 3 included additional adjustments to account for the glycated hemoglobin (HbA1c) level, body mass index (BMI), waist-to-hip ratio (WHR), systolic blood pressure (SBP), and central obesity category. The statistical results were presented as hazard ratios (HRs) and 95% confidence intervals (CIs).

#### 2.7.2 Analysis of the associations of metabolites with healthy lifestyles and age-related diseases

The original pool of 168 metabolites was analyzed to identify those associated with each age-related disease. Participants with missing baseline metabolomic data were excluded. Covariates with missing rates below 20% were imputed using multiple imputation. Metabolite values below the detection limit were replaced with half of the minimum detectable value. After natural logarithmic transformation [ln(x + 1)], metabolite concentrations were standardized to Z-scores.

For each disease cohort, 70% of participants were randomly assigned to the training set and 30% to the testing set. Age-related diseases were tested using a Cox regression model on the training set, with false discovery rate (FDR)–adjusted P-values calculated using the Benjamini–Hochberg method. Subsequently, a linear regression model was used to test whether a healthy lifestyle score was associated with each age-related disease, with multiple comparisons controlled at the 5% level using the Benjamini–Hochberg procedure.

Feature selection and interpretability analysis were conducted sequentially using extreme gradient boosting (XGBoost), Shapley additive explanation (SHAP), and Cox regression models. XGBoost builds an ensemble of decision trees by iteratively minimizing a regularized loss function, enabling the capture of complex nonlinear relationships while mitigating overfitting. The model was trained with a Cox proportional hazards objective, and early stopping based on the negative log partial likelihood was applied to determine the optimal number of boosting rounds. Key hyperparameters, including learning rate, tree depth, and regularization, were tuned using a held-out validation set. Model performance was evaluated using the concordance index (C-index), which quantifies the ability to correctly rank survival times. The XGBoost algorithm was applied to each cohort to identify the top metabolites among the previously significant ones, ranking features and reducing mutual interference.

To enhance interpretability, SHAP values were computed for the optimized XGBoost model. SHAP attributes each prediction’s deviation from the expected output to individual features, satisfying consistency and local accuracy. Global importance scores were obtained by averaging the absolute SHAP values across all training samples, producing a ranked panel of metabolites. The final list of the top ten metabolites for each disease was generated from the SHAP-based importance rankings.

#### 2.7.3 Assessment of mediation

To explain the associations between metabolites and age-related diseases, we further explored how a healthy lifestyle score affects the risk of each disease in the subsequent steps (R package “regmedint”). As a result of a healthy lifestyle, metabolite level improve, which, in turn, reduces the incidence of age-related diseases. In addition to assessing the mediating effects of the individual metabolites, we examined the joint effects of these metabolites. A notable feature of the mediation models was the adjustment for confounding variables, as in Model 3. We applied counterfactual mediation analysis to decompose the total effect of a healthy lifestyle score on incident age-related disease risk into natural direct and indirect effects. First, we fitted linear mediator models to estimate the effect of lifestyle scores on each metabolite, followed by fitting Cox proportional hazards models that simultaneously included exposure and mediator variables to quantify their joint association with disease risk. We then applied counterfactual mediation analysis to decompose the total effect into natural direct and indirect effects. This mediation framework relied on four key assumptions: no unmeasured confounding of the exposure–mediator, mediator–outcome, or exposure–outcome pathways was present and that any confounders of the mediator–outcome relationship were not themselves influenced by the exposure. This approach is grounded in the counterfactual (potential outcomes) framework for causal inference, which enables the formal decomposition of the total effect into natural direct and indirect effects under specified assumptions. Unlike traditional mediation methods, this framework allows for nonlinear and non-additive relationships between exposure, mediators, and outcomes. Specifically, the natural indirect effect is estimated by comparing the counterfactual outcome when the mediator is set to the value it would take under exposure versus under no exposure, while holding the exposure constant. The natural direct effect compares outcomes under different exposure levels, while fixing the mediator to its counterfactual value under no exposure. This decomposition provides interpretable estimates of the mediation pathways, facilitating mechanistic insights into how lifestyle factors influence disease risk through metabolic intermediates.

#### 2.7.4 Analyses of subgroups and sensitivity

Stratification by age, sex, and central obesity category was performed using multivariate Cox models. This study also underwent several sensitivity analyses to assess its robustness. By excluding participants who developed age-related diseases within the first two years of follow-up, the landmark analysis can be extended to reduce reverse causation bias; we excluded participants younger than 60 years to focus on the high-risk age group; we then recalculated the healthy lifestyle score using a leave-one-out method, sequentially omitting each lifestyle component to evaluate its independent contribution to the overall association.

Statistical significance was set at P < 0.05 with a 2-tailed test. Bonferroni corrections were applied during the analysis to compensate for the possibility of multiple testing errors. R version 4.2.1 was used for all the statistical analyses.

## 3 Results

### 3.1 Participants

**Figure 1:**
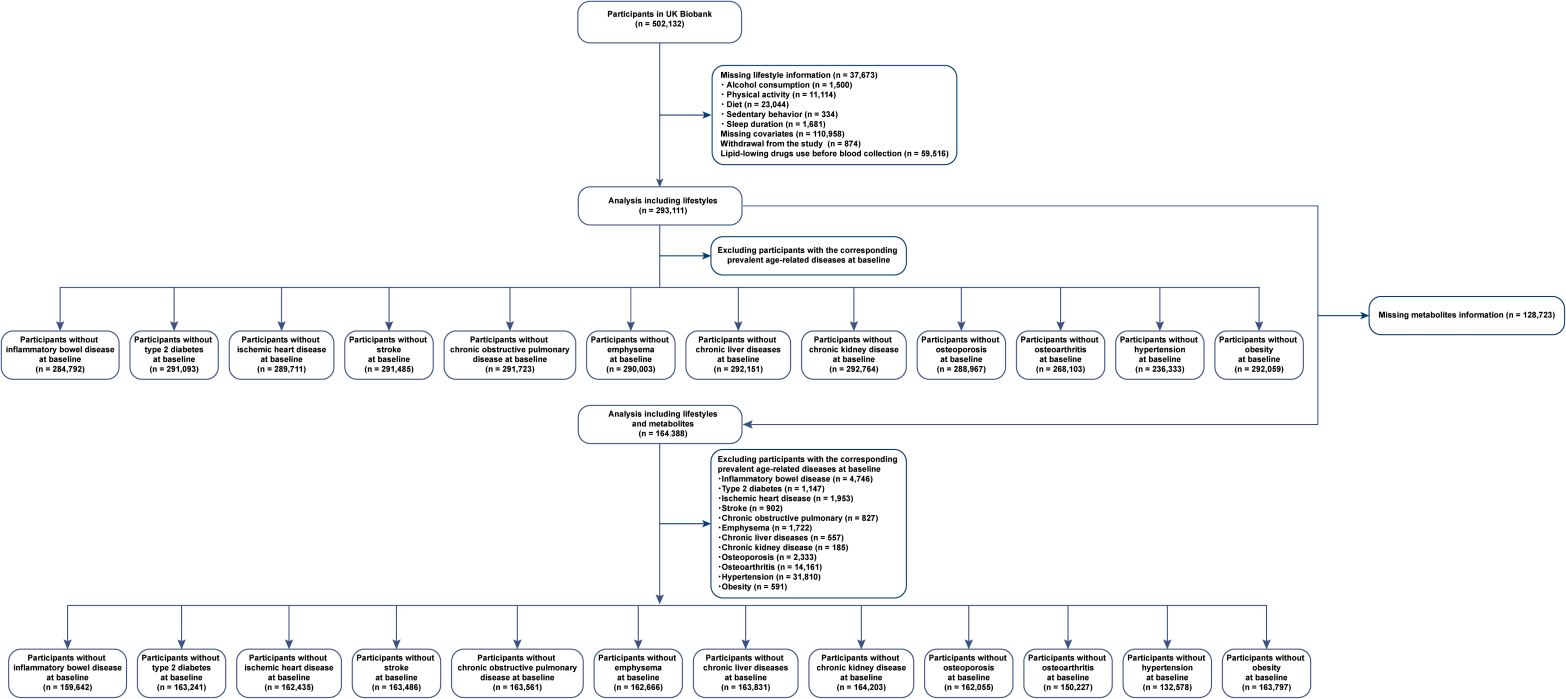
Participant selection flowchart.

A total of 37,673 individuals had no lifestyle information, 110,958 had no data for covariates, 874 withdrew from the study, and 59,516 had been using lipid-lowering drugs before blood collection. **Figure 1** illustrates our study design. The analysis of the association between healthy lifestyle and age-related diseases included 293,111 participants (females, 55%) with a median age of 56 years (interquartile range: 48, 62 years) at baseline.

After excluding individuals with missing metabolite data (n = 128,723), we included 164,388 participants (females, 55%) whose median age was 56 years (interquartile range: 48, 62 years) at baseline to identify metabolites reflecting healthy lifestyles and age-related diseases. Female participants, highly educated individuals, and households with high incomes were more likely to have healthy lifestyles. **Table 1** presents detailed statistics for individuals who were classified into the different healthy lifestyle categories. The presence of zeros in certain cells of **Table 1** reflects the categorical definition of lifestyle score used in this study. Participants classified as having a poor lifestyle (score 0–1) could only have 0–1 healthy lifestyle factors, those with an intermediate lifestyle (score 2–3) could only have 2–3 factors, and those with a healthy lifestyle (score 4–5) could only have 4–5 factors. As a result, other combinations are structurally absent due to the predefined categorization.

### 3.2 Disease incidence

The number of newly diagnosed cases for each disease varied depending on the number of participants included in each analysis. Participants with complete lifestyle data for newly diagnosed cases ranged from 1848 for emphysema to 3325 for hypertension, whereas participants with full lifestyle and metabolite data for newly diagnosed cases ranged from 1064 for emphysema to 19112 for hypertension. The details can be found in **Supplementary Tables 4**-**27**.

### 3.3 Lifestyle factors associated with age-related diseases

**Figure 2:**
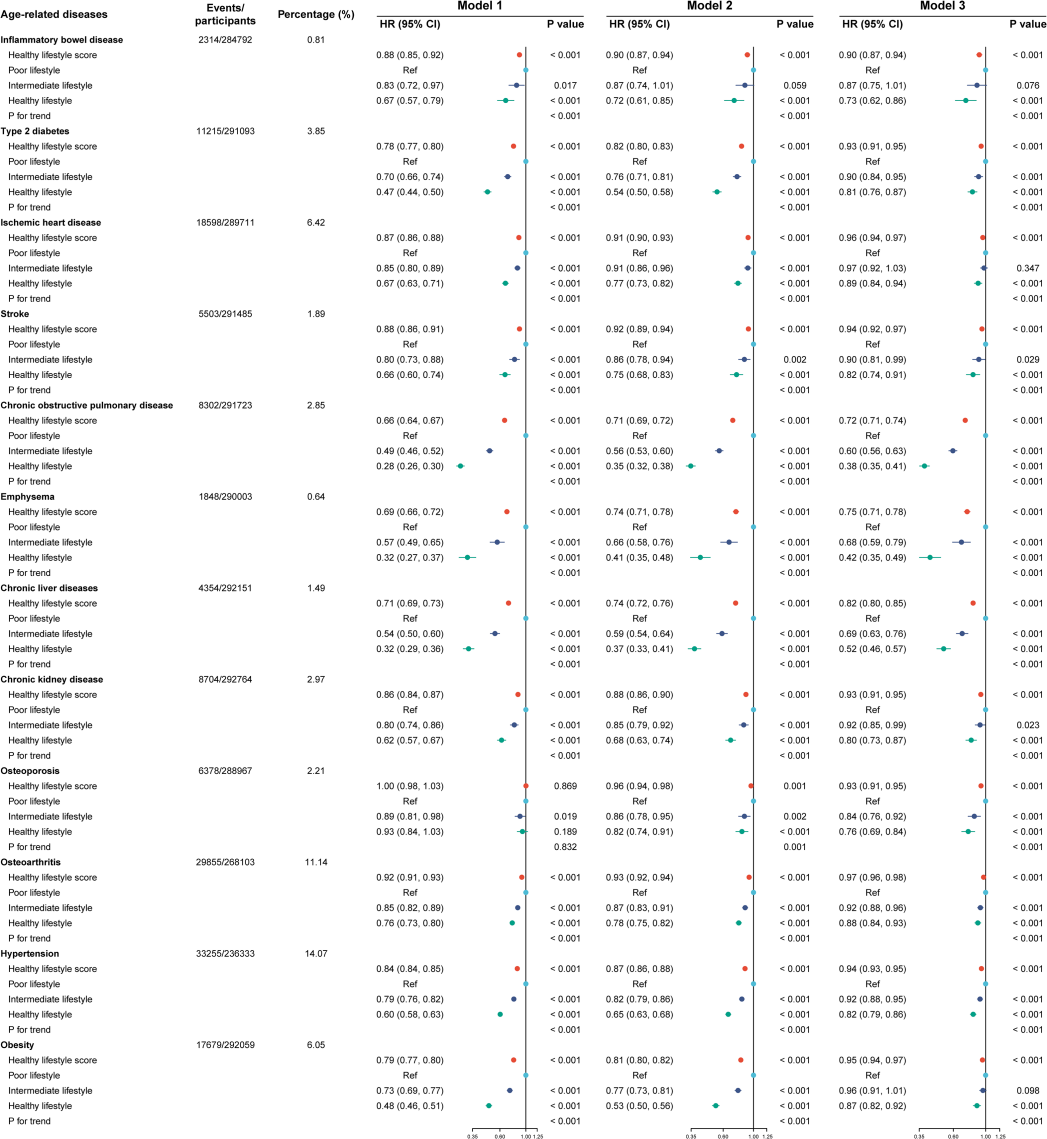
Lifestyle factors and the incidence of the 12 age-related diseases. Model 1 included no adjustments. Model 2 included adjustments for age, sex, racial or ethnic background, education, household income, and Townsend Deprivation Index. Model 3 included additional adjustments to account for the glycated hemoglobin level, body mass index, waist-to-hip ratio, systolic blood pressure, and central obesity category. HR. hazard ratio; CI, confidence interval. Cox regression was used to estimate HR and 95% CI in the testing set.

**Figure 2** shows that the three models adjusted for different covariates consistently showed that higher healthy lifestyle scores were associated with a lower risk of age-related diseases. In particular, healthy lifestyle scores contributed the most to preventing COPD (HR [95% CI]: 0.72 [0.71, 0.74]), followed by emphysema (HR [95% CI]: 0.75 [0.71, 0.78]).

The HR (95% CI) values for intermediate and healthy lifestyles in individuals diseases differed when scores were treated as categorical variables. Type 2 diabetes was associated with a HR (95% CI) of 0.90 (0.84, 0.95) and 0.81 (0.76, 0.87) for intermediate and healthy lifestyles, respectively. A summary of the HR (95% CI) values associated with individual diseases for intermediate and healthy lifestyles is provided below: 0.90 (0.81, 0.99) and 0.82 (0.74, 0.91) for stroke; 0.60 (0.56, 0.63) and 0.38 (0.35, 0.41) for COPD; 0.68 (0.59, 0.79) and 0.42 (0.35, 0.49) for emphysema; 0.69 (0.63, 0.76) and 0.52 (0.46, 0.57) for chronic liver diseases; 0.92 (0.85, 0.99) and 0.80 (0.73, 0.87) for CKD; 0.84 (0.76, 0.92) and 0.76 (0.69, 0.84) for osteoporosis; 0.92 (0.88, 0.96) and 0.88 (0.84, 0.93) for osteoarthritis; and 0.92 (0.88, 0.95) and 0.82 (0.79, 0.86) for hypertension. A significant *P_for trend_* (< 0.001) was observed for all 12 diseases associated with aging. Models 1 and 2 yielded similar results.

### 3.4 Components of the healthy lifestyle score and diseases associated with aging

A total of 51 significant associations out of 60 were noted, including 48 inverse associations (**Table 2**). Low alcohol consumption, less sedentary behavior, a healthy diet, physical activity, and sufficient sleep duration were associated with lower risks of most health problems associated with aging. The risk of osteoarthritis was higher in patients who engaged in high-intensity physical activity.

**Table 2:** Association between individual components of the healthy lifestyle and the risk of 12 age-related diseases.

Disease	Alcohol consumption		Physical activity		Diet		Sedentary behavior		Sleep duration	
	HR (95%*CI)*	*P*	HR (95%*CI)*	*P*	HR (95%*CI)*	*P*	HR (95%*CI)*	*P*	HR (95%*CI)*	*P*
Inflammatory bowel disease	1.04 (0.95, 1.14)	0.384	0.88 (0.80, 0.96)	0.004	0.87 (0.80, 0.95)	0.002	0.75 (0.65, 0.86)	< 0.001	0.84 (0.75, 0.95)	0.005
Type 2 diabetes	1.20 (1.15, 1.25)	< 0.001	0.83 (0.80, 0.86)	< 0.001	0.94 (0.90, 0.97)	0.001	0.81 (0.76, 0.88)	< 0.001	0.79 (0.75, 0.83)	< 0.001
Ischemic heart disease	1.06 (1.03, 1.10)	< 0.001	0.94 (0.91, 0.97)	< 0.001	0.94 (0.91, 0.96)	< 0.001	0.91 (0.86, 0.95)	< 0.001	0.86 (0.82, 0.90)	< 0.001
Stroke	0.95 (0.90, 1.01)	0.116	1.01 (0.95, 1.07)	0.7	0.92 (0.87, 0.97)	0.004	0.94 (0.85, 1.03)	0.161	0.83 (0.77, 0.89)	< 0.001
Chronic obstructive pulmonary disease	0.70 (0.67, 0.73)	< 0.001	0.74 (0.71, 0.78)	< 0.001	0.65 (0.62, 0.68)	< 0.001	0.76 (0.70, 0.83)	< 0.001	0.70 (0.66, 0.73)	< 0.001
Emphysema	0.78 (0.71, 0.86)	< 0.001	0.69 (0.63, 0.76)	< 0.001	0.65 (0.59, 0.72)	< 0.001	0.80 (0.68, 0.94)	0.008	0.77 (0.68, 0.86)	< 0.001
Chronic liver diseases	0.78 (0.73, 0.83)	< 0.001	0.82 (0.77, 0.87)	< 0.001	0.88 (0.83, 0.94)	< 0.001	0.81 (0.72, 0.91)	< 0.001	0.70 (0.65, 0.76)	< 0.001
Chronic kidney disease	1.24 (1.18, 1.30)	< 0.001	0.84 (0.81, 0.88)	< 0.001	0.88 (0.85, 0.92)	< 0.001	0.86 (0.79, 0.93)	< 0.001	0.77 (0.72, 0.81)	< 0.001
Osteoporosis	1.02 (0.97, 1.08)	0.444	0.83 (0.79, 0.88)	< 0.001	0.99 (0.94, 1.05)	0.797	0.98 (0.91, 1.06)	0.607	0.77 (0.72, 0.83)	< 0.001
Osteoarthritis	0.89 (0.87, 0.91)	< 0.001	1.08 (1.05, 1.11)	< 0.001	1.01 (0.99, 1.04)	0.234	0.92 (0.88, 0.96)	< 0.001	0.87 (0.84, 0.90)	< 0.001
Hypertension	0.97 (0.95, 0.99)	0.013	0.88 (0.86, 0.90)	< 0.001	0.97 (0.95, 0.99)	0.008	0.91 (0.87, 0.94)	< 0.001	0.88 (0.85, 0.90)	< 0.001
Obesity	0.98 (0.95, 1.01)	0.216	0.98 (0.95, 1.01)	0.117	0.95 (0.92, 0.98)	0.002	0.89 (0.84, 0.94)	< 0.001	0.86 (0.83, 0.90)	< 0.001

Model 3 included adjustments for all variables results. HR. hazard ratio; CI, confidence interval. Cox regression was used to estimate HR and 95% CI in the testing set.

### 3.5 Metabolite profiles associated with healthy lifestyles and age-related diseases

The Cox regression results for each age-related disease are presented in **Supplementary Table 28**. Detailed results of the linear regression for metabolites and healthy lifestyle scores are provided in **Supplementary Table 29**. **Supplementary Figure 1** shows the top metabolites identified by XGBoost that had the greatest relevance to each age-related disease along with the top 10 metabolites identified by SHAP. For example, 132 metabolites were significantly correlated with type 2 diabetes. The association between healthy lifestyle scores and 119 metabolites was particularly strong. On the basis of the XGBoost algorithm, the top 10 metabolites were identified using SHAP analysis based on the XGBoost results.

**Figure 3:**
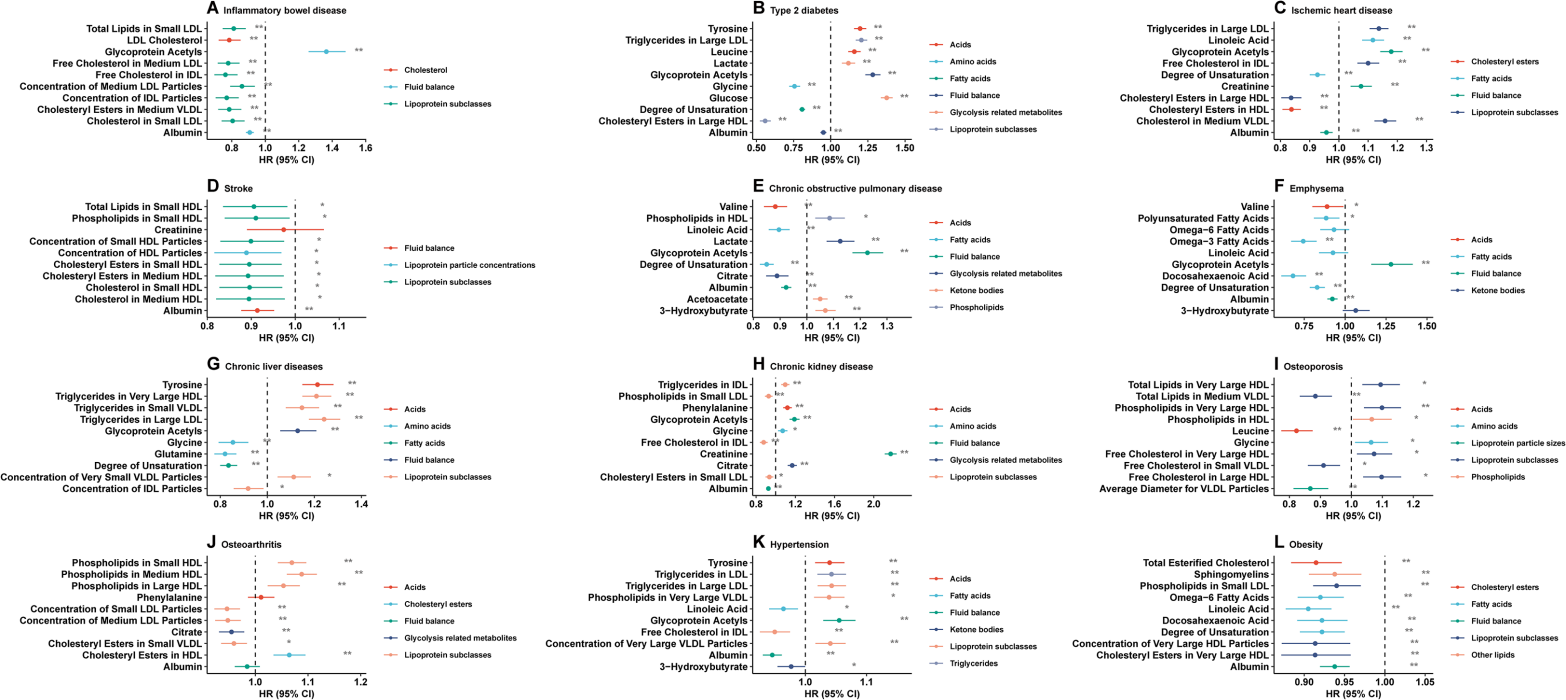
Healthy lifestyle score and incidence of the following age-related diseases are associated with the top ten identified metabolites. **(A)** inflammatory bowel disease, **(B)** type 2 diabetes, **(C)** ischemic heart disease, **(D)** stroke, **(E)** chronic obstructive pulmonary disease, **(F)** emphysema, **(G)** chronic liver diseases, **(H)** chronic kidney disease, **(I)** osteoporosis, **(J)** osteoarthritis, **(K)** hypertension, **(L)** obesity. HR hazard ratio, CI confidence interval. Model 3 included adjustments for all variables. Cox regression was used to estimate HR and 95% CI in the testing set.

As shown in **Supplementary Figure 1**, we identified the top 10 metabolites associated with each age-related disease. Various metabolic pathways involved these metabolites (such as fatty acids, total lipids, other lipids, fluid balance, glycolysis-related metabolites, and lipoprotein subclasses). A Cox regression model was used for the training set to analyze the association between metabolites and age-related disease risks in **Figure 3**. Detailed HR values for the 10 metabolites and 12 age-related diseases are presented in **Supplementary Table 30**. Significant associations were observed between most of these metabolites and age-related diseases.

**Figure 3** illustrates the negative associations between the identified metabolites and the risks of IBD, stroke, emphysema, and obesity. Half of the identified metabolites were negatively associated with other age-related diseases.

As shown in **Figure 3**, most of the identified metabolites were negatively associated with the risk of IBD, stroke, emphysema, and obesity. In contrast, half of the identified metabolites were negatively associated with the risk of other age-related diseases. A significant inverse association was found between albumin levels and IBD, diabetes type 2, ischemic heart disease, stroke, COPD, emphysema, CKD, hypertension, and obesity, while chronic liver disease and IBD were also significantly inversely correlated with the intermediate-density lipoprotein (IDL) particle concentration.

### 3.6 Metabolites’ mediating roles

**Figure 4:**
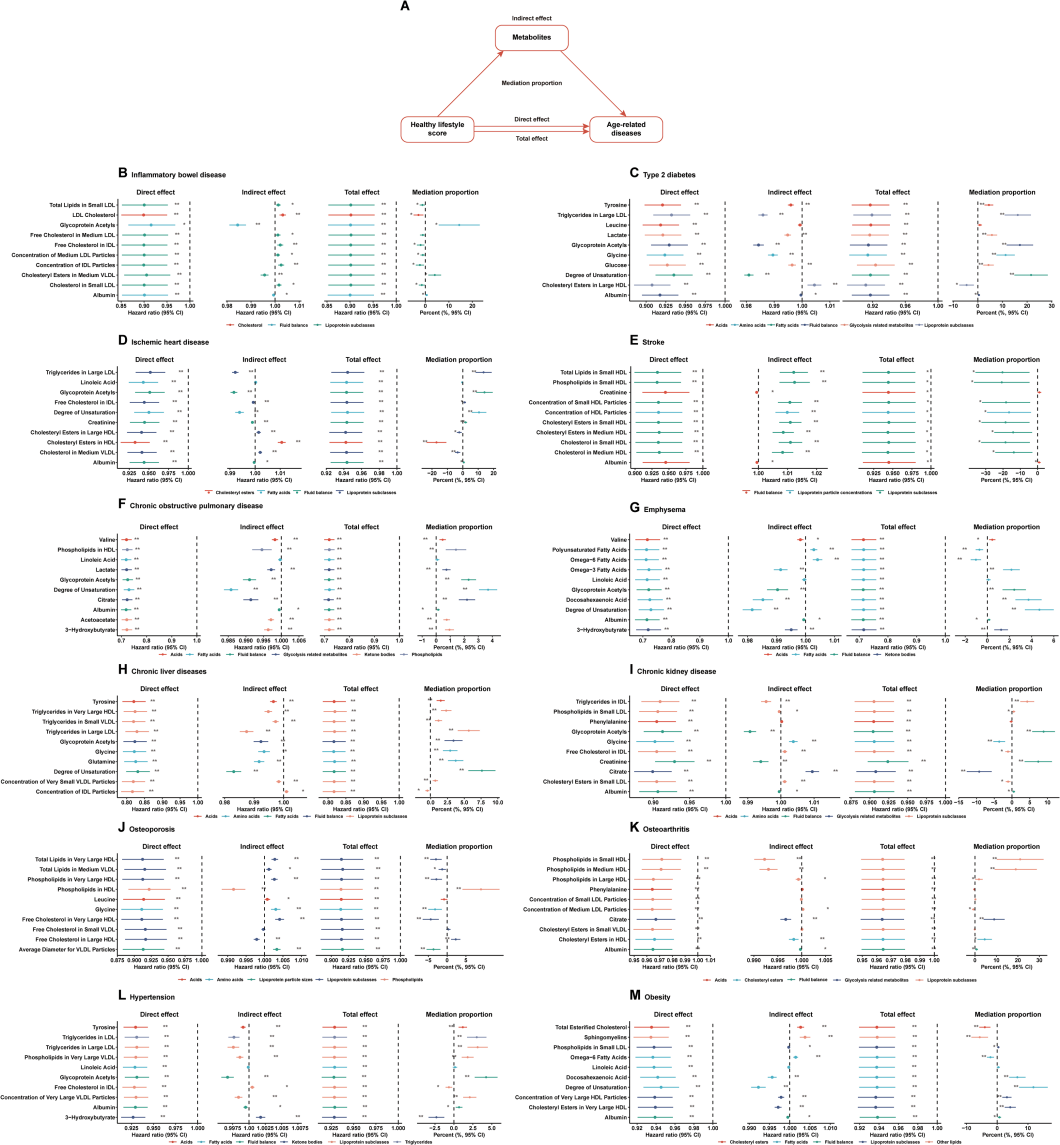
Healthy lifestyle score and incident age-related diseases: multiple mediation analysis with metabolites. **(A)** Plot of the mediation effect of each of the top ten metabolites on the healthy lifestyle score and the incidence of age-related diseases, **(B)** inflammatory bowel disease, **(C)** type 2 diabetes, **(D)** ischemic heart disease, **(E)** stroke, **(F)** chronic obstructive pulmonary disease, **(G)** emphysema, **(H)** chronic liver diseases, **(I)** chronic kidney disease, **(J)** osteoporosis, **(K)** osteoarthritis, **(L)** hypertension, and **(M)** obesity. HR, hazard ratio; CI, confidence interval. Model 3 included adjustments for all variables results. Cox regression was used to estimate HR and 95% CI in the testing set.

Our mediation analysis revealed that the identified metabolites mediated the associations between the healthy lifestyle score and age-related diseases, either individually or jointly. The results of the mediation analysis are presented in **Figure 4**; **Supplementary Table 31**. A significant, natural, and indirect association between healthy lifestyle scores and age-related diseases was observed for most of the selected metabolites. For example, glycoprotein acetylation contributed 14.43% of the overall association between healthy lifestyle scores and IBD, whereas the low-density lipoprotein (LDL) cholesterol level masked it by 2.92%. The fatty acid content based on the degree of unsaturation showed a 21.64% contribution to the association between the healthy lifestyle score and type 2 diabetes, while 4.57% of the association was masked by the presence of cholesterol esters in large high-density lipoprotein (HDLs). Importantly, the mediation results from models 1 and 2, in which potential mediators were deliberately excluded from the adjustment set, were consistent with those of Model 3 (**Supplementary Tables 32**, **33**). This consistency suggests that our findings are robust and not driven by overadjustment bias.

### 3.7 Analyses of subgroups and sensitivity

The association between healthy lifestyle scores and the risk of each age-related disease stratified by age, sex, and central obesity is shown in **Supplementary Figure 2**. A consistent association was observed between the healthy lifestyle score and a reduced risk of each age-related disease and its subtypes across various groups.

Despite excluding individuals those who developed age-related diseases within two years of follow-up (**Supplementary Figure 3**) and individuals aged less than 60 years (**Supplementary Figure 4**), the findings remained robust. Similar results were observed when each healthy lifestyle component was excluded to derive a new healthy lifestyle risk score (**Supplementary Figures 5**-**9**).

## 4 Discussion

Our prospective cohort study of a large UK Biobank population revealed that higher scores for a healthy lifestyle were associated with a lower risk of 12 age-related diseases. A healthy lifestyle score was also associated with robustness and consistency across 12 age-related diseases. Ten metabolites were associated with each age-related disease, and most of them were significantly correlated with all age-related diseases. Our mediation analysis revealed that several of the identified metabolites appeared to mediate each age-related disease, with the effect mediated by the healthy lifestyle score. Our findings provide a more comprehensive understanding of the preventative potential of specific healthy lifestyle behaviors and the mediating role of metabolites in these effects.

The findings showed that the risk of the 12 age-related diseases was negatively correlated with the healthy lifestyle score, indicating that a healthy lifestyle provided protection against the 12 age-related diseases by reducing alcohol consumption, improving diet patterns, enhancing physical activity, ensuring sufficient sleep, and decreasing sedentary behaviors.

Previous studies have identified correlations between metabolomics data and healthy lifestyles, providing insights into the intricate relationships between individual metabolic processes and behaviors, including diet, physical activity, alcohol consumption, and sleeping patterns (41–44). A recent study identified 83 metabolites that could represent healthy lifestyle behaviors and showed a significant impact of these metabolites on the incidence of dementia (41). In other studies, healthy lifestyle-related metabolites have been associated with significant reductions in the incidence of coronary artery disease (29, 45). A study involving approximately 80,000 participants identified 81 metabolites that reflected a healthy lifestyle and indicated a lower risk of rheumatoid arthritis (46). Owing to the limited sample size and short follow-up durations of previous studies on other age-related diseases (not mentioned above), our study provides additional evidence for the benefits of a healthy lifestyle score in preventing these diseases. However, these studies did not account for lifestyle factors or did not establish metabolites as mediators. Furthermore, metabolic research, which focuses on small molecules within cells, tissues, or organisms, has become increasingly relevant for research on biological aging. Chronological age is associated with the levels of most metabolites, and many nonlinear relationships have been observed (47). Similar to our study, numerous lipid-related metabolites have been identified that may be crucial for neuronal function.

We identified 10 metabolites associated with 12 age-related diseases. Among these metabolites, albumin, a recognized diagnostic marker for in various diseases, including 10 diseases associated with aging in the UK Biobank, was the most significant, followed by glycoprotein acetylation. Ten age-related diseases showed significant positive associations with albumin levels, while eight showed significant positive associations with glycoprotein acetyl levels. Interestingly, our study underscores the clinical significance of albumin and glycoprotein acetyls, which are both recognized as having diagnostic power for a wide range of diseases in the UK Biobank (48–51). Our study indicated that albumin levels, which were previously shown to contribute significantly to the diagnosis and treatment of various diseases (51, 52), are positively correlated with the risk of age-related diseases. Albumin, the most abundant plasma protein, plays a multifaceted role in maintaining colloidal osmotic pressure, transporting endogenous and exogenous molecules, and exerting antioxidant and anti-inflammatory effects (53, 54). Beyond its structural abundance, albumin participates in redox regulation through its free thiol group, which can undergo reversible oxidation in response to oxidative stress (53, 54). Alterations in albumin structure and function have been implicated in various chronic conditions, including liver disease and metabolic syndromes (53, 54). Because of their intricate role in disease pathogenesis, especially in the aging population, glycoprotein acetyls have attracted considerable attention in the clinical realm. Glycoprotein acetyls, for instance, have been identified as biomarkers of inflammation, which promotes insulin resistance (55). Specifically, glycoprotein acetylation represents a composite NMR signal derived from the N-acetyl methyl groups of multiple acute-phase glycoproteins. Rather than reflecting a single metabolite, glycoprotein acetyls capture systemic inflammation and are robustly associated with cardiometabolic risk and all-cause mortality (56–58). Their stability and broad inflammatory coverage make them a clinically relevant marker for chronic low-grade inflammation, particularly in aging populations (56–58). Furthermore, we found that linoleic acid, free cholesterol, and triglycerides in large LDLs, as well as the degree of unsaturation, were associated with most age-related diseases. In addition to showing promise as a diagnostic assay in primary care, the assessment of metabolic signatures may indicate shared metabolic pathways underlying disease progression (59–62). Our study revealed intriguing associations between metabolites and specific age-related diseases, providing insights into the potential mechanisms of specific age-related diseases. On the basis of these findings, tailored therapeutic interventions and precision medicine strategies could be developed for specific age-related diseases.

Our study had several strengths. We demonstrated a consistently and significantly lower risk of 12 age-related diseases in individuals with higher healthy lifestyle scores. The results of our study underscore the importance of maintaining a healthy lifestyle to reduce the risk of these 12 age-related diseases. Thus, lifestyle interventions are essential for reducing the burden of age-related diseases in the population. The second unique aspect of this study is the identification of specific metabolites as potential mediators of the beneficial effects of healthy lifestyles on most age-related diseases. On the basis of this finding, biomarkers or screening tools can be developed to assess the risk of age-related diseases by identifying metabolites associated with healthy lifestyles. In terms of early intervention and personalized medicine, this may be of great clinical benefit. Third, we ensured the statistical power of our model and the reliability of our results through a substantial study population. Sensitivity analyses were conducted to verify the robustness and validity of our conclusions.

This study had several limitations. First, inferring causal relationships was beyond the scope of the current study. Reverse causality between diet patterns and type 2 diabetes cannot be ruled out, because type 2 diabetes may influence diet patterns. Second, although lifestyle factors may have been miscategorized owing to their self-reported nature, the instruments used were validated (e.g., food frequency and physical activity questionnaires). Furthermore, prior research in the UK Biobank showed a high degree of consistency between self-reported data and information from primary care records, indicating the accuracy of self-reported data (42). Although comprehensive, the methods for measuring metabolites were neither error-free nor errorless. Third, despite taking steps to control for various confounding variables, the results may still have been biased by unaccounted or residual confounding variables. Fourth, despite identifying a metabolic signature associated with healthy lifestyles and a reduced risk of 12 age-related diseases, the specific metabolic mechanisms underlying these associations require further assessment and validation. Fifth, data pertaining to lifestyle factors such as alcohol intake were collected from the questionnaires but were not based on objective markers of alcohol consumption. Thus, recall and misclassification biases for these variables cannot be ruled out. Last, the volunteer-based recruitment process creates a “healthy volunteer bias” where participants are generally healthier, more health-conscious, and of higher socioeconomic status than the general UK population, which may have limited the generalizability of research findings from the study. While this bias may have influenced the external validity of the data, the consistency of the findings with other studies suggests that exposure-disease associations in the UK Biobank are often widely generalizable.

## 5 Conclusions

The conclusions of our study support the association between improved adherence to healthy lifestyles and significantly reduced incidence of12 age-related diseases. Additionally, we identified 10 metabolites associated with the risk of 12 age-related diseases, proving that the 12 age-related diseases were associated with the same metabolic pathways. Our study, identified the individual and joint mediating roles of these metabolites in 12 age-related diseases. Our findings provide new insights into the complex relationship between healthy lifestyles, metabolites, and diseases, and have implications for developing targeted interventions to prevent and manage age-related diseases.

## Data Availability

The original contributions presented in the study are included in the article/**Supplementary Material**. Further inquiries can be directed to the corresponding author.
